# Associations of Serum Per‐ and Polyfluoroalkyl Substances (PFAS) With Epigenetic Age and DNA Methylation in US Airport Firefighters

**DOI:** 10.1002/em.70085

**Published:** 2026-10-09

**Authors:** Lauren M. Ward, Jaclyn M. Goodrich, Miriam M. Calkins, Shawn C. Beitel, Antonia M. Calafat, Alberto J. Caban‐Martinez, Paola Louzado‐Feliciano, Julianne Cook Botelho, Melissa Furlong, Randy Krause, Rob Matthis, Jeff Hughes, Jefferey L. Burgess

**Affiliations:** ^1^ Department of Environmental Health Sciences University of Michigan School of Public Health Ann Arbor Michigan USA; ^2^ Division of Environmental Natural Sciences Duke University Durham North Carolina USA; ^3^ Department of Community, Environment and Policy University of Arizona Mel and Enid Zuckerman College of Public Health Tucson Arizona USA; ^4^ National Center for Environmental Health, Centers for Disease Control and Prevention Atlanta Georgia USA; ^5^ Department of Public Health Sciences University of Miami Miller School of Medicine Miami Florida USA; ^6^ Port of Seattle Fire Department SeaTac Washington USA; ^7^ Port of Portland Airport Fire and Rescue Portland Oregon USA; ^8^ Orange County Fire Authority Irvine California USA

**Keywords:** epigenetics, fire service, immunotoxicity, occupational health

## Abstract

Per‐ and polyfluoroalkyl substances (PFAS) are synthetic chemicals used in a variety of consumer and industrial applications for their water‐ and oil‐resistant properties. Firefighters can be exposed to PFAS as part of their job through gear, aqueous film‐forming foams (AFFFs) used to extinguish aircraft and liquid fuel fires, along with exposure through other combustible materials during fire suppression activities. PFAS exposure has been linked to a variety of health conditions including cancer, obesity, reproductive issues, and immunosuppression. Epigenetic modifications may be one mechanism underlying PFAS toxicity. In this study, we used epigenetic indicators to assess association of PFAS serum concentrations with (1) epigenetic aging and (2) DNA methylation at loci within genes related to immune function. Blood leukocyte DNA methylation was quantified via the Infinium EPIC array from 184 participants in the Fire Fighter Cancer Cohort Study involved in aircraft rescue and firefighting, and serum PFAS was measured via on‐line solid phase extraction liquid chromatography isotope dilution tandem mass spectrometry. We observed inverse associations between PFAS concentrations and epigenetic clocks including between serum linear perfluorooctanoic acid (n‐PFOA) with four epigenetic clocks (*p* < 0.05); however, most did not remain significant in sensitivity analyses. Associations (*q* < 0.1) were observed between perfluorodecanoic acid, n‐PFOA, sum of branched perfluorooctane sulfonic acid isomers and differential methylation of specific loci within 34 immune‐related genes. Further research can help to better understand the functional impacts of the observed differential methylation on immune health.

## 1 Introduction

Per‐ and polyfluoroalkyl substances (PFAS) are a class of synthetic chemicals that have oil and water‐resistant properties and have thus been used in a variety of consumer and industrial products since the 1940s. Due to the chemical structure of PFAS, which contain strong carbon‐fluorine bonds, this class of chemicals breaks down slowly in the environment. Some PFAS bioaccumulate in human tissues with repeated environmental exposures (Batzella et al. 2024; Li et al. 2022). Firefighters may be exposed to PFAS from multiple sources in their work including burning materials, turnout gear, aqueous film‐forming foams (AFFFs), and contamination of air, dust, and water at fire scenes and the workplace (Mazumder et al. 2023; Conner et al. 2026). Firefighters have been reported to have elevated serum concentrations of some long‐alkyl chain PFAS when compared to the general population or workers in other occupations (Trowbridge et al. 2020; Lucas et al. 2022). Many fire departments in the United States have transitioned away from PFAS‐containing foams, but this transition is still in progress for aircraft firefighting (Federal Aviation Administration 2025). Class B firefighting foams, designed to extinguish liquid fuel fires such as aircraft fires, can contain the long‐alkyl chain PFAS perfluorooctane sulfonic acid (PFOS) and perfluorooctanoic acid (PFOA) (Council, ITR 2023). In addition to use during an active fire, AFFFs are sometimes used in training exercises, though this practice has decreased in recent years (Council, ITR 2023). Firefighters may be occupationally exposed to PFAS through dermal or inhalation routes, or by ingesting contaminated food and water (Mazumder et al. 2023; Conner et al. 2026; Rosenfeld et al. 2022; Hernandez‐Fajardo et al. 2025; Herkert et al. 2025).

PFAS exposure has been associated with adverse health impacts, including increased risk for several cancers, reproductive consequences, poor developmental and birth outcomes, metabolic dysfunction, high cholesterol, thyroid disease, ulcerative colitis, and immunosuppression (Sunderland et al. 2019; DeWitt et al. 2019; DeWitt et al. 2024; International Agency for Research on Cancer 2025; Ehrlich et al. 2023). AFFFs have demonstrated immunotoxic effects resulting in impaired antibody production in mouse studies and in children (Grandjean et al. 2012; McDonough et al. 2020). Probable links have been established between PFAS and various chronic health conditions as well as between PFOS and PFOA with cancer risk (Sunderland et al. 2019; International Agency for Research on Cancer 2025).

Epigenetic age is a biomarker of aging that uses epigenetic marks, typically DNA methylation, to approximate biological age (Oblak et al. 2021). Epigenetic age acceleration has been linked to cancers, mortality (Fransquet et al. 2019), aging‐related cognitive outcomes (Jain et al. 2022), and declining cardiovascular health (Joyce et al. 2021). Epigenetic age is calculated with established algorithms based on DNA methylation at specific sets of loci, called epigenetic clocks. In the Fire Fighter Cancer Cohort Study (FFCCS), we previously reported associations between several PFAS and accelerated epigenetic aging among municipal firefighters (Goodrich et al. 2021) and accelerated epigenetic age in incumbent firefighters compared to recruits (Nwanaji‐Enwerem et al. 2023).

Epigenetic modifications are potential mechanisms of PFAS toxicity (Kim et al. 2021). DNA methylation at specific loci changes with age, but exposures to toxicants, environmental factors, and diseases can also result in modifications to the epigenome. Previous research in a cell line has reported increased global DNA methylation associated with PFOS and PFOA, which may lead to silencing of tumor suppressor genes (Pierozan et al. 2024). A 2022 study of adolescents in Ronneby, Sweden who were exposed to PFAS via AFFF‐contaminated water observed that blood DNA methylation at 364 cytosine‐guanine dinucleotides (CpG sites) was associated with exposure (Xu et al. 2022). A prior study of adult women in the same cohort identified 117 differentially methylated CpG sites in blood (Xu et al. 2020). The differentially methylated sites in both studies were annotated to genes involved in cell signaling and metabolism, and health endpoints including cancer and endocrine disorders (Xu et al. 2020) (Xu et al. 2022). Another study of Dutch men identified 38 differentially methylated regions (DMRs) in blood associated with PFOS exposure (van den Dungen et al. 2017). Our previous work among municipal firefighters in the FFCCS found associations between the sum of branched isomers of PFOA (Sb‐PFOA), linear PFOS (n‐PFOS), perfluorononanoic acid (PFNA), perfluorodecanoic acid (PFDA), perfluoroundecanoic acid (PFUnDA) serum concentrations and differentially methylated positions and regions in blood, including in genes involved in immune activation and inflammatory response (Goodrich et al. 2021).

Considering that aircraft rescue and firefighting (ARFF) personnel may be exposed to greater amounts of PFAS from AFFFs, in addition to other common exposures among all firefighters and background exposures from their daily environment (Calkins et al. 2026), it is important to understand the influence of PFAS exposure on their health. In this study, we utilize epigenetic indicators to assess associations between PFAS and epigenetic aging and DNA methylation of target genes related to immune function. We hypothesized that serum PFAS concentrations would be associated with accelerated epigenetic aging and that there would be an association between elevated concentrations of serum PFAS and differential DNA methylation at loci on genes with biological functions related to immunity in ARFF.

## 2 Materials and Methods

### 2.1 Study Population

Study participants were recruited as part of the FFCCS, a prospective cohort study involving fire departments across the United States. The FFCCS was initiated in 2016 with the goal of collecting and analyzing data on exposures and various health endpoints from 10,000 US firefighters over the course of 30 years with a particular focus on cancer risk (Burgess et al. 2025). Inclusion criteria for this analysis of a subset of the FFCCS included being an active‐duty ARFF with available data on serum PFAS, reported chronological age, and blood leukocyte DNA methylation. All participants provided informed consent. All study procedures were approved by the institutional review boards of the University of Arizona and the University of Miami.

### 2.2 Data and Sample Collection

Participants were administered a questionnaire at the time of study enrollment to collect demographic, occupational, and health information. Blood was collected by a phlebotomist for PFAS quantification (serum) and DNA isolation. Blood samples were stored temporarily at −20°C, then stored at −80°C until use. Sample collection procedures were previously described (Goodrich et al. 2021; Burgess et al. 2025).

### 2.3 PFAS Quantification

Frozen serum samples were shipped overnight on dry ice to the CDC National Center for Environmental Health. Serum concentrations of nine PFAS (n‐PFOS, linear PFOA [n‐PFOA], PFDA, perfluorohexane sulfonic acid (PFHxS), sum of perfluoromethylheptane sulfonic acid isomers (Sm‐PFOS), PFNA, PFUnDA, Sb‐PFOA, and 2‐(N‐methyl‐perfluorooctane sulfonamido) acetic acid (MeFOSAA)) were quantified via on‐line solid phase extraction liquid chromatography isotope dilution tandem mass spectrometry (Calkins et al. 2026; Burgess et al. 2023; Nematollahi et al. 2023). The limit of detection (LOD) for all PFAS was 0.1 ng/mL. Concentrations below the LOD were replaced with LOD/sqrt(2) for statistical analysis. PFHxS, n‐PFOS, Sm‐PFOS and n‐PFOA were detected in all samples. PFNA was detected in 98.4% of samples, PFDA in 93.4%, and PFUnDA in 82.6%. MeFOSAA was detected in 29.9% of samples. Sb‐PFOA was excluded from further analysis because of its low detection frequency (1.1%). These nine PFAS measures comprised the CDC panel used for this study and were selected to facilitate comparison with prior structural‐firefighter studies and NHANES population biomonitoring data.

### 2.4 EPIC Array and Calculation of Epigenetic Age

DNA methylation was quantified at > 850,000 CpG sites via the Infinium Methylation EPIC array at the University of Michigan Advanced Genomics Core using methods previously described (Goodrich et al. 2021). Briefly, 500 ng of DNA isolated from blood leukocytes was bisulfite converted for downstream Infinium array analysis using Zymo kits. DNA methylation was quantified via the EPIC array (Moran et al. 2016). Raw data were processed using the R packages minfi to read raw image files and ENmix for quality control and normalization (Xu et al. 2017; Aryee et al. 2014). Probes were excluded if undetected in ≥ 5% of samples, cross‐reactive, located on sex chromosomes, or overlapping SNPs. Background and dye bias correction were performed using noob and regression on logarithm of internal control, respectively, followed by quantile normalization (Xu et al. 2017). Surrogate variable analysis (Teschendorff et al. 2011) on control probe intensities identified three principal components explaining the technical variance in the data, which were included as covariates to account for batch effects.

Epigenetic age was approximated with the DNA methylation data via seven clocks (Horvath, Hannum, PhenoAge, Skin‐Blood, GrimAge, DunedinPACE, and telomere length—DNAmTL). Among the first generation clocks which were trained to predict chronological age, the Hannum clock is sensitive to variations in the environment, while the Horvath clock is a stable representation of the cellular aging process across multiple tissues (Gibson et al. 2019). The second generation clocks, including PhenoAge and GrimAge, were trained on health indicators, which strengthens their ability to predict lifespan and health outcomes (Levine et al. 2018; Lu, Quach, et al. 2019). Telomere length, which is known to shorten with age, is another indicator of biological aging that can be estimated with DNA methylation data (Lu, Seeboth, et al. 2019). Epigenetic age indicators were calculated via software developed by Horvath (2013). The EPIC data was uploaded to the New Methylation Age Calculator (hosted at https://dnamage.clockfoundation.org) to calculate six biomarkers of epigenetic age, and DunedinPACE was calculated via the R package “DunedinPACE” (Belsky et al. 2022). For all clocks except DNAmTL, we calculated epigenetic age acceleration (EAA) by regressing the clock estimates of age on chronological age and computing the residual values. A positive residual value indicates accelerated epigenetic aging compared to chronological age. These residuals were used as the outcome variables in downstream statistical analyses. Calculated telomere length measurements were used directly in models as the outcome. Proportions of CD4^+^ T cells, CD8+ T cells, natural killer cells, B cells, monocytes, and granulocytes in each sample were estimated from the DNA methylation data using an established algorithm designed for Infinium data (Horvath 2013) along with relative abundance of plasmablasts, CD8^+^CD28^−^CD45RA^−^ T cells, naive CD4^+^ T cells, and naive CD8^+^ T cells.

### 2.5 Immune Gene Inclusion Criteria

A literature search was conducted to identify immune genes that had previously been associated with PFAS exposure. The search was conducted in PubMed and the PFAS‐Tox Database. Inclusion criteria included published articles reporting at least one PFAS of interest (n‐PFOS, n‐PFOA, PFUnDA, PFDA, PFNA, MeFOSAA, PFHxS, Sb‐PFOA, Sm‐PFOS) significantly associated with gene expression or DNA methylation of individual genes with an immunity‐related function. Due to variations in statistical methods, the authors' most stringent cutoff for statistical significance in each study was used. Only studies conducted in humans or in vitro experiments using human cells were included. Ten studies were identified that met these criteria (Table S1). The search strategy is detailed in Figure S1. DNA methylation data at all available loci covered by the EPIC array for each of the 126 unique genes identified from these articles were used in final analyses. The final dataset consisted of 3856 CpG sites within these genes.

### 2.6 Statistical Analysis

All statistical analyses were performed in the R Project for Statistical Computing, version 4.3.1 or greater. A sample of 184 airport firefighters in the FFCCS were eligible for inclusion in this analysis (Figure S2). Descriptive statistics were calculated for covariates including demographic and occupational variables, PFAS serum concentrations, and epigenetic age biomarkers. PFAS concentrations were natural‐log transformed to achieve a normal distribution. Confounders were determined by examining relationships between ln‐transformed PFAS and covariates (age, sex, race, ethnicity, smoking history, BMI, fire department, years of firefighting, EPIC array‐batch variables, estimated blood cell type proportions, and estimated relative cell abundances). Pearson's correlations, two‐sample *t*‐tests, and ANOVA tests were performed to examine relationships between ln‐transformed PFAS and continuous, dichotomous, and multiple‐category variables, respectively. Welch's *t*‐test was used when sample variances were unequal. Cell‐type covariates were selected separately for the epigenetic age and immune gene analyses. For epigenetic age models, selection was based on correlations between estimated cell types and the epigenetic indicators. For immune gene models, selected cell types explained overall variation in the CpG methylation data.

#### 2.6.1 Epigenetic Age Analysis

The six EAA variables and DNAmTL were each regressed on the eight evaluated PFAS measures. Seven PFAS detected in > 80% of samples were modeled as continuous concentrations (natural log‐transformed) while MeFOSAA was dichotomized as detected versus not detected because its detection frequency was < 30%. Sb‐PFOA was not evaluated in these analyses due to low detection frequency (1.1%). After running unadjusted analyses, the model was adjusted for potential confounders: age, sex, race and ethnicity, smoking status, EPIC array batch, and cell types (neutrophils, monocytes, natural killer cells, CD8^+^CD28^−^CD45RA^−^ T cells, CD4^+^ naive T‐cells, CD8^+^ naive T‐cells, CD4^+^ T‐cells, and plasmablasts). We also ran the same model without cell types included. *p*‐values < 0.05 were considered statistically significant. Bonferroni‐adjusted *p*‐values were evaluated at < 0.05 across 63 comparisons: eight individual PFAS measures and one NASEM summed‐PFAS category, each tested against seven epigenetic indicators. This corresponds to an unadjusted *p*‐value threshold of 0.05/63 (approximately 0.000794).

The National Academies of Sciences, Engineering, and Medicine published guidelines in 2022 for patient follow‐up after PFAS serum testing (National Academies of Sciences, Engineering and Medicine 2022). These follow‐up guidelines set forth specific recommendations for three ranges of summed concentrations of seven PFAS (MeFOSAA, PFHxS, PFOA, PFDA, PFUnDA, PFOS, PFNA) in serum or plasma (< 2 ng/mL, 2 to < 20 ng/mL, and ≥ 20 ng/mL). The NASEM categories were modeled as an additional exposure.

A sensitivity analysis was conducted after removing four data points that were identified as potentially influential by Cook's distance. The Cook's distance threshold for potentially significant points was 0.0241, calculated via the formula 4/n‐number of model coefficients. Points were removed if they were potentially influential in ≥ 20% of exposure‐outcome models across multiple epigenetic age indicators and had a measurement for at least one epigenetic age indicator that fell more than three standard deviations away from the mean.

#### 2.6.2 Immune Gene Analysis

We included 3856 CpG sites annotated to 126 immune genes previously associated with PFAS exposure as identified by our literature search. Linear regression models were used to model the relationships between beta values representing the proportion methylated for each of the CpG sites with each PFAS. The primary model included adjustment for EPIC array batch and the potential confounders age, sex, race/ethnicity, smoking status, military service, and cell type estimates (CD4+ T cells, CD8^+^ T cells, neutrophils, monocytes, and B cells). We additionally ran a model excluding cell type adjustment. We considered associations with a false discovery rate‐adjusted *p*‐value (*q*‐value) < 0.1 to be statistically significant (Benjamini and Hochberg 1995).

## 3 Results

### 3.1 Study Population Characteristics

The study sample included 184 incumbent ARFF firefighters enrolled in 2019 and 2020 from six fire departments in the US states of Alaska, Arizona, California, Florida, Oregon, and Washington. Table 1 displays descriptive statistics for the study population. Demographic characteristics of the study sample did not differ significantly from the demographics of all ARFF participants in the FFCCS. Firefighters participating in this analysis were primarily male (88.6%) with an average chronological age of 43 years. The mean length of firefighting career was 15 years. Forty‐four participants (23%) had served as active‐duty members of the military. The majority of participants reported their race as non‐Hispanic White (68.5%), followed by Hispanic (10.9%), and non‐Hispanic Black (6.0%). Self‐reported races outside of these three categories were aggregated into “Other” (14.7%) to maintain anonymity of participants. Most participants had never been smokers (89.1%).

**TABLE 1:** Age, occupational, and demographic variables for the ARFF sample (*n* = 184).

Age and epigenetic age		Mean (SD)
Chronological age (years)		43.1 (11.0)
Hannum DNAm Age‐residual		0.21 (3.81)
Horvath DNAm Age‐residual		0.38 (4.38)
SkinBlood Clock‐residual		0.00 (3.68)
DNAm PhenoAge‐residual		−0.21 (5.04)
DNA GrimAge‐residual		−0.94 (2.75)
DunedinPACE score		0.88 (0.11)
DNAmTL (Telomere length)		7.31 (0.27)
Firefighting Experience		*n* (%)
Career years^a^		15.4 (10.8)
Volunteer years^a^		2.0 (2.9)
Demographic and additional covariates		
BMI^a^	Normal (18.5–24.9 kg/m^2^)	41 (25.0)
	Overweight (25–29.9)	78 (47.6)
	Obese (30+)	45 (27.4)
Smoking status	Never smoker	164 (89.1)
	Former	17 (9.2)
	Current	0 (0.0)
	Status unknown	3 (1.6)
Race/Ethnicity	Hispanic	20 (10.9)
	Non‐Hispanic Black	11 (6.0)
	Non‐Hispanic White	126 (68.5)
	Other	27 (14.7)
Sex	Female	21 (11.4)
	Male	163 (88.6)
Previous Active‐duty military service	Yes	44 (23.9)
	No	140 (76.1)

*Note:* A total of 183 reported career years of firefighting and 177 volunteer years; 164 had BMI data; 181 reported smoking status.

^a^ Several participants were missing data.

### 3.2 PFAS Exposure Assessment

Seven PFAS, n‐PFOS, n‐PFOA, PFDA, PFHxS, Sm‐PFOS, PFNA, and PFUnDA, were detected in more than 80% of participants' serum samples. MeFOSAA was detected in 29.9% of participants. Serum PFAS concentrations are presented in Table 2. The 2022 NASEM guidelines indicate that there is a potential for adverse health outcomes, especially in sensitive populations, with summed serum concentrations of seven PFAS from 2 to < 20 ng/mL and an increased risk of adverse health outcomes at ≥ 20 ng/mL (National Academies of Sciences, Engineering and Medicine 2022). All firefighters in the sample had summed serum PFAS concentrations ≥ 2 ng/mL and 8.2% had concentrations ≥ 20 ng/mL (Table 3).

**TABLE 2:** PFAS serum concentrations in ARFF study participants (*n =* 184) (2019–2020).

PFAS	Detection frequency (%)	Geometric mean, ng/mL (95% CI)	25th percentile, 75th percentile (ng/mL)
PFHxS	100	1.90 (1.70, 2.11)	1.20, 2.63
n‐PFOS	100	3.23 (2.95, 3.51)	2.08, 4.60
Sm‐PFOS	100	1.95 (1.77, 2.15)	1.40, 3.20
n‐PFOA	100	1.35 (1.26, 1.45)	1.00, 1.80
Sb‐PFOA	1.1	^a^	^a^
PFNA	98.4	0.43 (0.39, 0.46)	0.30, 0.60
PFDA	93.5	0.18 (0.17, 0.20)	0.10, 0.30
PFUnDA	82.6	0.14 (0.13, 0.16)	0.10, 0.20
MeFOSAA	29.9	< LOD (< LOD, 0.10)	< LOD, 0.10

*Note:* The limit of detection (LOD) was 0.1 ng/mL for all PFAS.

^a^ Not calculated due to low detection.

**TABLE 3:** NASEM guideline categories of summed PFAS concentrations.

Serum PFAS sum^a^	*n* (%)
< 2 ng/mL	0 (0.0)
2 to < 20 ng/mL	169 (91.9)
≥ 20 ng/mL	15 (8.2)

^a^ Sum of MeFOSAA, PFHxS, linear and branched PFOA, linear and branched PFOS, PFNA, PFDA, and PFUnDA.

n‐PFOS, PFNA, PFDA, PFHxS, and Sm‐PFOS were detected at higher concentrations in males than females (ANOVA; *p* < 0.05). n‐PFOA, n‐PFOS, PFNA, PFDA, PFHxS, and Sm‐PFOS were significantly positively correlated with age and/or length firefighting career (Pearson correlation; *r* > 0.30) (Figure S3).

### 3.3 Associations Between PFAS Serum Concentrations and Epigenetic Age Indicators

In adjusted linear regression models, there were few statistically significant associations between PFAS and epigenetic age biomarkers. Inverse associations were observed between n‐PFOA concentrations and four clocks (Horvath, Hannum, PhenoAge, and Skin‐Blood) (*p* < 0.05). A significant inverse association was observed between PFUnDA and the Hannum clock (*p* = 0.05) (Table 4). Only the association between n‐PFOA and the Skin‐Blood clock remained statistically significant after Bonferroni correction in the primary analysis (adjusted *p* = 0.0315; Table 4). The NASEM sum PFAS categories were not associated with any epigenetic age indicators.

**TABLE 4:** Associations between PFAS measures and epigenetic age indicators (*n* = 181).

PFAS		Epigenetic age indicator						
		Horvath	Hannum	PhenoAge	SkinBlood	GrimAge	Dunedin PACE	Telomere length
PFHxS	Estimate (SE)	0.10 (0.47)	0.08 (0.35)	0.22 (0.51)	−0.27 (0.38)	0.22 (0.22)	0.02 (0.01)	0.007 (0.01)
	*p*‐value	0.82	0.82	0.66	0.48	0.32	0.12	0.63
	Bonferroni‐adjusted *p*‐value	1.00	1.00	1.00	1.00	1.00	1.00	1.00
n‐PFOS	Estimate (SE)	0.31 (0.61)	0.16 (0.46)	0.11 (0.67)	0.71 (0.50)	0.18 (0.29)	0.02 (0.01)	−0.0004 (0.02)
	*p*‐value	0.61	0.74	0.86	0.16	0.53	0.16	0.98
	Bonferroni‐adjusted *p*‐value	1.00	1.00	1.00	1.00	1.00	1.00	1.00
Sm‐PFOS	Estimate (SE)	−0.22 (0.57)	−0.11 (0.43)	0.35 (0.63)	0.09 (0.47)	0.23 (0.27)	0.02 (0.01)	0.02 (0.02)
	*p*‐value	0.70	0.80	0.58	0.84	0.41	0.13	0.34
	Bonferroni‐adjusted *p*‐value	1.00	1.00	1.00	1.00	1.00	1.00	1.00
n‐PFOA	Estimate (SE)	−2.00 (0.66)	−1.10 (0.50)	−1.43 (0.73)	−1.90 (0.53)	0.07 (0.32)	−0.004 (0.02)	0.009 (0.20)
	*p*‐value	0.003	0.03	0.05	0.0005	0.83	0.79	0.66
	Bonferroni‐adjusted *p*‐value	0.189	1.00	1.00	0.0315	1.00	1.00	1.00
PFNA	Estimate (SE)	−0.04 (0.63)	−0.47 (0.47)	0.18 (0.69)	0.26 (0.52)	0.13 (0.30)	−0.002 (0.01)	−0.006 (0.02)
	*p*‐value	0.95	0.32	0.80	0.62	0.66	0.89	0.75
	Bonferroni‐adjusted *p*‐value	1.00	1.00	1.00	1.00	1.00	1.00	1.00
PFDA	Estimate (SE)	−0.32 (0.63)	−0.23 (0.47)	−0.51 (0.69)	0.05 (0.51)	−0.03 (0.30)	−0.009 (0.01)	−0.01 (0.02)
	*p*‐value	0.61	0.63	0.46	0.92	0.91	0.55	0.60
	Bonferroni‐adjusted *p*‐value	1.00	1.00	1.00	1.00	1.00	1.00	1.00
PFUnDA	Estimate (SE)	−0.37 (0.60)	−0.90 (0.45)	−0.80 (0.66)	−0.22 (0.49)	0.14 (0.29)	−0.02 (0.01)	−0.01 (0.02)
	*p*‐value	0.54	0.05	0.22	0.66	0.63	0.18	0.44
	Bonferroni‐adjusted *p*‐value	1.00	1.00	1.00	1.00	1.00	1.00	1.00
MeFOSAA	Estimate (SE)	0.14 (0.70)	0.08 (0.53)	0.69 (0.77)	−0.12 (0.57)	−0.38 (0.33)	0.006 (0.02)	0.02 (0.02)
	*p*‐value	0.84	0.88	0.37	0.83	0.26	0.72	0.26
	Bonferroni‐adjusted *p*‐value	1.00	1.00	1.00	1.00	1.00	1.00	1.00
NASEM sum ≥ 20 ng/mL	Estimate (SE)	0.46 (1.15)	−0.30 (0.87)	−0.96 (1.26)	−0.24 (0.94)	−0.51 (0.55)	0.006 (0.03)	−0.008 (0.03)
	*p*‐value	0.69	0.73	0.45	0.80	0.36	0.83	0.82
	Bonferroni‐adjusted *p*‐value	1.00	1.00	1.00	1.00	1.00	1.00	1.00

Adjusted for age, sex, race and ethnicity, smoking status, batch, and estimated cell type proportions for neutrophils, monocytes, NK cells, CD8 + CD28‐CD45RA‐ cells, CD4+ naive T‐cells, CD8+ naive T‐cells, CD4+ T‐cells, and plasmablasts. Models with a *p*‐value < 0.05 are bolded and in red text. Bonferroni adjustment includes 63 comparisons; adjusted *p*‐values < 0.05 indicate significance after correction. Continuous PFAS measures were natural‐log transformed; MeFOSAA was modeled as detected versus not detected, and the NASEM category as ≥ 20 versus 2 to < 20 ng/mL.

A sensitivity analysis was conducted after removing four participants identified as influential points (Table S2). Of the four epigenetic clocks associated with n‐PFOA concentrations in the adjusted model, only the association with the Skin‐Blood indicator retained nominal significance in the sensitivity analysis (*p* = 0.03). The inverse association between PFUnDA and the Hannum indicator also retained nominal significance (*p* = 0.04). Neither sensitivity‐analysis association would meet the same Bonferroni criterion.

### 3.4 Immune Gene DNA Methylation Analysis

Three PFAS were associated with differential DNA methylation at specific loci at a q‐value < 0.1. Sm‐PFOS, n‐PFOA, and PFDA were associated with DNA methylation at 53 unique CpG sites (Table 5). Sm‐PFOS was inversely associated with one CpG site within *MCF2L2*. n‐PFOA was associated with methylation levels at 17 loci across 14 genes. PFDA was associated with 45 loci across 31 genes.

**TABLE 5:** Associations between serum PFAS concentrations and DNA methylation in immune genes (loci significant at *q* < 0.1 are shown).

PFAS	Probe ID	Gene name	Locus	Effect estimate (SE)^a^, ^b^	*p*	*q*
n‐PFOA	cg03237507	*DDX60*	chr4: 169241417	−0.0262 (0.0062)	0.00004	0.0598
	cg17748182	*DVL1*	chr1: 1277441	−0.0069 (0.0017)	0.0001	0.0598
	cg21191843	*EBF1*	chr5: 158153361	0.0085 (0.0022)	0.0002	0.0598
	cg24337359	*FAT1*	chr4: 187630465	0.0046 (0.0012)	0.0002	0.0598
	cg12692682	*INTS7*	chr1: 212202711	0.0052 (0.0014)	0.0002	0.0598
	cg25304359	*MCF2L2*	chr3: 183075480	−0.0169 (0.0045)	0.0002	0.0598
	cg24192846	*PPP1R11*	chr6: 30037773	−0.0497 (0.0126)	0.0001	0.0598
	cg02918557	*RADIL*	chr7: 4901337	0.0338 (0.009)	0.0002	0.0598
	cg23572228		chr7: 4923575	0.0081 (0.0019)	0.00005	0.0598
	cg06971903	*RASGRP3*	chr2: 33720546	−0.0292 (0.0071)	0.00006	0.0598
	cg07827972	*SCARB1*	chr12: 125288649	0.0207 (0.0051)	0.00007	0.0598
	cg04141261		chr12: 125308274	−0.0157 (0.0041)	0.0002	0.0598
	cg13802355	*SLC11A1*	chr2: 219246070	0.0616 (0.0171)	0.0004	0.0980
	cg17078326	*STX18*	chr4: 4509338	−0.0179 (0.0046)	0.0001	0.0598
	cg02951344	*SYTL2*	chr11: 85522279	0.0173 (0.0046)	0.0003	0.0606
	cg06177974		chr11: 85522300	0.026 (0.0069)	0.0002	0.0598
	cg10791260	*TNFSF15*	chr9: 117568588	0.0049 (0.0012)	0.0001	0.0598
Sm‐PFOS	cg25304359	*MCF2L2*	chr3: 183075480	−0.0145 (0.0032)	0.00001	0.0508
PFDA	cg08889593	*ADAR*	chr1: 154568347	−0.0204 (0.0042)	0.000003	0.0030
	cg27530370		chr1: 154589288	0.0071 (0.002)	0.0006	0.0648
	cg15499799	*ADORA2A*	chr22: 24823110	−0.0137 (0.0036)	0.0002	0.0427
	cg15517631	*ANKRD40*	chr17: 48785189	0.0022 (0.0006)	0.0007	0.0689
	cg14224638	*C1S*	chr12: 7171203	−0.0554 (0.0115)	0.000003	0.0030
	cg17563034	*CYTL1*	chr4: 5021268	−0.02 (0.0053)	0.0003	0.0427
	cg03237507	*DDX60*	chr4: 169241417	−0.0252 (0.0058)	0.00002	0.0100
	cg13494355	*DVL1*	chr1: 1274420	−0.007 (0.0019)	0.0003	0.0427
	cg21191843	*EBF1*	chr5: 158153361	0.0077 (0.0021)	0.0003	0.0427
	cg17847344		chr5: 158478734	0.0417 (0.0098)	0.00003	0.0120
	cg07256113		chr5: 158526263	0.0037 (0.0011)	0.0008	0.0728
	cg24376311	*HLA‐DQB1*	chr6: 32632788	0.0283 (0.007)	0.00007	0.0158
	cg17534770	*IGSF11*	chr3: 118753871	−0.002 (0.0005)	0.0004	0.0470
	cg05401880	*IL12RB1*	chr19: 18209690	−0.0096 (0.0026)	0.0003	0.0427
	cg03410961	*IRF4*	chr6: 391680	0.0123 (0.0029)	0.00005	0.0142
	cg21249230	*LINC01405*	chr12: 111374363	−0.021 (0.0059)	0.0005	0.0561
	cg26666444	*LRBA*	chr4: 151238143	−0.0173 (0.005)	0.0007	0.0689
	cg12164564		chr4: 151368765	−0.0101 (0.0028)	0.0003	0.0427
	cg02580005		chr4: 151504187	0.0058 (0.0017)	0.0009	0.0804
	cg17967817	*MCF2L2*	chr3: 183072580	−0.0117 (0.0026)	0.00001	0.0076
	cg10833439	*MX1*	chr21: 42793818	0.0046 (0.0014)	0.001	0.0964
	cg23256466	*NFKB2*	chr10: 104159199	0.0225 (0.0053)	0.00004	0.0127
	cg04498739	*PDK4*	chr7: 95226308	−0.0051 (0.0014)	0.0005	0.0553
	cg24192846	*PPP1R11*	chr6: 30037773	−0.0547 (0.0116)	0.000005	0.0031
	cg23572228	*RADIL*	chr7: 4923575	0.0074 (0.0018)	0.00007	0.0158
	cg06971903	*RASGRP3*	chr2: 33720546	−0.0357 (0.0064)	0.00000009	0.0003
	cg00955769	*RNF39*	chr6: 30042385	−0.0059 (0.0015)	0.00009	0.0177
	cg16170495		chr6: 30042626	0.0446 (0.0121)	0.0003	0.0427
	cg05853632		chr6: 30043273	0.0085 (0.0019)	0.00002	0.0100
	cg07827972	*SCARB1*	chr12: 125288649	0.0176 (0.0048)	0.0003	0.0427
	cg07690796		chr12: 125301012	−0.0197 (0.0054)	0.0004	0.0440
	cg04141261		chr12: 125308274	−0.0157 (0.0038)	0.00005	0.0142
	cg12079885		chr12: 125328475	0.0121 (0.0028)	0.00003	0.0109
	cg09689279	*SHC4*	chr15: 49169965	−0.006 (0.0015)	0.0001	0.0234
	cg20665205		chr15: 49254494	−0.0054 (0.0016)	0.0008	0.0709
	cg13802355	*SLC11A1*	chr2: 219246070	0.0737 (0.0156)	0.000005	0.0031
	cg16851467	*STX18*	chr4: 4427496	−0.0178 (0.0049)	0.0003	0.0427
	cg09380927		chr4: 4506904	0.007 (0.002)	0.0007	0.0689
	cg10513480	*SUPT16H*	chr14: 21852135	0.0279 (0.0076)	0.0003	0.0427
	cg06717083	*SUSD4*	chr1: 223462267	0.0103 (0.0028)	0.0003	0.0427
	cg26349554	*SYTL2*	chr11: 85522295	0.0037 (0.0011)	0.0007	0.0689
	cg06177974		chr11: 85522300	0.0315 (0.0062)	0.000001	0.0022
	cg17205461	*TAX1BP1*	chr7: 27779758	0.0022 (0.0005)	0.00007	0.0158
	cg13854854	*TCF4*	chr18: 52975755	0.0072 (0.0021)	0.0005	0.0584
	cg06627114		chr18: 53090930	−0.0102 (0.0025)	0.00006	0.0155

*Note:* results are ordered by PFAS and then gene name (not by statistical significance).

^a^ Effect estimate represents the proportion of methylation changes per ln‐transformed unit of PFAS (ng/mL).

^b^ Model adjusted for age, sex, race/ethnicity, smoking status, military service, batch variables, and cell type estimates (CD4+ T cells, CD8+ T cells, neutrophils, monocytes, and B cells).

PFDA, n‐PFOA, and Sm‐PFOS were collectively associated with 53 unique CpG sites across 34 genes. PFDA, n‐PFOA, and Sm‐PFOS were all associated with hypomethylation of loci on *MCF2L2*. Sm‐PFOS and n‐PFOA were both inversely associated with DNA methylation at the same site (probe ID cg25304359), while PFDA was inversely associated with methylation at another site in the same gene (probe ID cg17967817). Both PFDA and n‐PFOA were associated with DNA methylation in the genes *DDX60, DVL1, EBF1, PPP1R11, RADIL, RASGRP3, SCARB1, SLC11A1, STX18*, and *SYTL2*. There was a positive association between both PFDA and n‐PFOA concentrations at one site (probe ID cg06177974) in the *SYTL2* promoter. The 10 CpG sites associated with two PFAS each, together with gene functions, are summarized in Table S3.

## 4 Discussion

In this cross‐sectional study of ARFF US firefighters, we observed few statistically significant associations between serum concentrations of PFAS and biomarkers of epigenetic aging. We observed significant associations between serum concentrations of three long‐alkyl chain PFAS (PFOA, Sm‐PFOS, PFDA) and differential DNA methylation at 53 unique CpG sites spanning 34 immune genes.

All firefighters included in this analysis had summed serum concentrations of seven PFAS ≥ 2 ng/mL, the lowest threshold set by the 2022 NASEM guidelines to inform clinical care of exposed patients. Fifteen firefighters (8.2%) had summed PFAS concentrations ≥ 20 ng/mL, which was of comparable magnitude to the 9% estimated by NASEM for the US population represented by NHANES 2017–2018 in this unadjusted comparison (National Academies of Sciences, Engineering and Medicine 2022). Previous research in the FFCCS reported elevated PFHxS and Sm‐PFOS serum concentrations of firefighters compared to 2015–2016 NHANES (Burgess et al. 2023), which is also reflected in the current subset of ARFF. PFAS concentrations from our study were previously compared to NHANES serum concentrations among adults from a similar time period (2017–2020). In adjusted analyses, PFHxS, Sm‐PFOS, and PFUnDA were significantly higher among ARFF firefighters (Calkins et al. 2026). Differences in age, sex, and other population characteristics limit this comparison, which does not establish equivalent exposure distributions or health risks. Direct comparisons with other firefighter cohorts are limited because studies often report individual PFAS concentrations rather than the proportion at or above the NASEM summed‐PFAS threshold.

A 2018–2019 study of 799 Australian aviation rescue firefighters found that firefighters had mean serum concentrations of PFOS, perfluoroheptane sulfonate (PFHpS), and PFHxS higher than the general population. The 2013–2014 measurements of the same group observed PFOS and PFHxS concentrations six to ten times higher than the general population. However, firefighters who began work after PFAS‐containing foams were replaced with a fluoro‐surfactant‐free alternative in 2010 due to the implementation of new regulations had serum concentrations similar to members of the general population (Nilsson et al. 2022). Alternative fluorine‐free foams have been introduced in the US in recent years. The Department of Defense published a set of guidelines in 2023 requiring that proposed fluorine‐free foams may not contain more than 1 part per billion (i.e., 1 ng/mL) of PFAS, while continuing to meet performance benchmarks (Defense, USDO 2023). Our samples were collected in 2019–2020. This cross‐sectional analysis did not evaluate changes before and after replacement of PFAS‐containing materials and cannot establish whether phaseout efforts explain the concentrations observed in this cohort.

We observed inverse associations between two PFAS and four epigenetic aging indicators at the nominal significance threshold; two associations retained nominal significance in the sensitivity analysis. These predominantly inverse findings did not support our original hypothesis of accelerated epigenetic aging with higher serum PFAS concentrations. Our previous work in structural firefighters in the FFCCS found significant accelerations in epigenetic aging associated with PFHxS, n‐PFOA, and Sm‐PFOS (Goodrich et al. 2021). While we also observed accelerated epigenetic age in incumbent structural firefighters compared to recruits in the FFCCS, we reported that years of service were associated with decelerations in epigenetic age among incumbents (Nwanaji‐Enwerem et al. 2023). Differences in occupational co‐exposures, PFAS exposure profiles, demographic characteristics, clock construction, and covariate adjustment may contribute to the divergent findings. Sampling variability and sensitivity to influential observations may also contribute. These are possible explanations that the present study cannot distinguish. There have been mixed results among the few studies evaluating PFAS exposure and epigenetic aging. A pilot study of 59 adult women in Ronneby, Sweden found that elevated PFAS serum concentration from contaminated drinking water was not associated with accelerated epigenetic aging according to the Horvath clock, though the same concentrations were associated with differential DNA methylation (Xu et al. 2020). A second study from the same research team examined DNA methylation and epigenetic aging in 63 children from the same area echoed these findings (Xu et al. 2022). A study that examined NHANES data from 1999 through 2018 observed similar associations between PFOS, PFOA, and PFNA serum concentrations and decelerations in another measure of biological aging based on nine blood‐based biomarkers—Phenotypic Age Acceleration—in adult men and women. Similar to the present study, PFOA was most consistently associated with decelerated biological aging (Chaney and Wiley 2023). An NHANES study found few statistically significant associations between serum PFAS concentrations and epigenetic clocks with the exception of a positive association between PFNA and GrimAge (Acquaah and Obeng‐Gyasi 2026). Taken together, these studies support associations between some PFAS and DNA methylation at specific loci, while evidence for associations with epigenetic aging is inconsistent. Inverse associations with epigenetic indicators do not establish a protective effect of PFAS or the absence of adverse health effects.

Building on studies in firefighters and other populations (Goodrich et al. 2021; van den Dungen et al. 2017; Petroff et al. 2023; Schrott et al. 2025), we report associations between some long‐alkyl chain PFAS (n‐PFOA, Sm‐PFOS, and PFDA) with DNA methylation at 53 CpG sites within 34 immune‐related genes. We used a hypothesis‐driven approach focusing on 126 immune‐related genes that had been associated with at least one PFAS in previous studies of gene expression or epigenetic regulation. DNA methylation at four CpG sites within *SCARB1* was associated with n‐PFOA or PFDA. *SCARB1* encodes a receptor that mediates viral entry into cells. It is also a plasma membrane receptor for high‐density lipoprotein cholesterol (Gracia‐Rubio et al. 2026) and could also be relevant for the relationships between PFAS and elevated cholesterol (Haug et al. 2023).

Eleven additional genes had multiple differentially methylated sites (*DVL1*, *EBF1*, *LRBA*, *MCF2L2*, *RADIL*, *RNF39*, *SHC4*, *SLC11A1*, *STX18*, *SYTL2*, *TCF4*). *STX18*, *SYTL2*, *RNF39*, and *EBF1*, which play roles in immune cell signaling and antiviral response, were differentially methylated at 3 CpG sites each. *EBF1* is involved in B cell differentiation and signaling relevant to adaptive immunity (Li et al. 2024). *SYTL2* is associated with NK cell activation and signaling‐ integral components of the innate immune system (Bezman et al. 2012; Chen et al. 2022). n‐PFOA, PFDA, and Sm‐PFOS concentrations were associated with DNA methylation at two sites within *MCF2L2*. *MCF2L2* in cord blood from the Norwegian BratMat cohort was identified as associated with both PFAS and episodes of the common cold in childhood by Pennings et al. (2016). *MCF2L2* was hypermethylated in children exposed to AFFF in drinking water in a study in Sweden (Xu et al. 2022). Further research is needed to investigate whether *MCF2L2* expression or functionality may be impacted by AFFF‐associated PFAS and whether this is linked to immunotoxicity.

Nine CpG sites in *EBF1*, *RADIL*, *SCARB1*, *SYTL2*, *DDX60*, *PPP1R11*, *RASGRP3*, and *SLC11A1* were associated with both n‐PFOA and PFDA. Together with the *MCF2L2* site associated with n‐PFOA and Sm‐PFOS, these constitute the 10 shared loci in Table S3, which summarizes functions of genes containing loci associated with more than one PFAS. The RNA helicase encoded by *DDX60* functions as an antiviral factor (Miyashita et al. 2011) and is involved in downstream cell signaling that results in the production of interferons and inflammatory cytokines (Onomoto et al. 2021). *PPP1R11* has been proposed to control T cell activation (Joshi et al. 2019) and to moderate the inflammatory response to bacterial infections (McKelvey et al. 2016). The protein encoded by *RASGRP3* limits the production of proinflammatory interleukins in macrophages (Tang et al. 2014) and mediates the activation of signaling pathways that are over‐activated in some human cancers where the gene is over‐expressed (Nagy et al. 2014). These results suggest that exposure to PFOA and PFDA may be associated with differential methylation of several genes involved in the innate immune system.

PFDA was associated with differential methylation at the greatest number of loci. This is consistent with our previous FFCCS study where PFDA was associated with hypomethylation at 50 CpG sites, and hypermethylation at five sites (at *q* < 0.2) among structural firefighters in an epigenome‐wide analysis (Goodrich et al. 2021). PFDA plasma concentration during the first trimester of pregnancy was also previously associated with altered DNA hydroxymethylation and methylation in cord blood (Petroff et al. 2023), suggesting PFDA is an epigenotoxicant even at relatively low concentrations. PFDA is found in various household textiles like carpet, furniture, and leather, as well as in other products such as food packaging and waterproofing sprays (Kaiser et al. 2024). While other PFAS demonstrated a downward trend in NHANES data collected between 1999 and 2018, serum geometric mean concentrations of PFDA increased 24% in males and 27% in females between 2015 and 2018, although concentrations were relatively low, only about twice the LOD (Sonnenberg et al. 2023). The US Food and Drug Administration (FDA) banned the use of PFOS and PFOA in food packaging in 2016, and PFDA may have been one of the replacements used instead (64). PFDA is associated with altered liver biomarkers and hepatotoxicity, immunosuppression, developmental outcomes, spontaneous abortion, and male and female reproductive impacts in animal models (Kaiser et al. 2024). Further research is needed on PFDA and its long‐term health impacts.

Several strengths and limitations exist in the present study. This study uses comprehensive measures—epigenetic age and DNA methylation—to individually assess the impacts of eight PFAS. The cross‐sectional design cannot establish the temporal ordering of PFAS exposure and epigenetic differences or support causal conclusions. In terms of other limitations, first, the study population is primarily white, non‐Hispanic, and male, so information on PFAS impacts in diverse populations may not be captured. The inclusion of data on physical fitness and general health may help to clarify any differences in overall health status between structural and ARFF firefighters that may potentially attenuate the effects of PFAS exposure. Second, firefighters are occupationally exposed to multiple toxicants, in addition to PFAS, that were not measured here. Third, the exact source of PFAS exposures in this cohort was not evaluated and may include AFFFs, other workplace sources, and environmental exposures outside of the workplace. Fourth, markers of immune function were not measured directly, and this is an area for future research. Lastly, these findings have not yet been replicated in an independent cohort, which limits our ability to assess their reproducibility and generalizability to other firefighter or occupational populations.

## 5 Conclusions

This research assessed associations between serum concentrations of eight PFAS with epigenetic age and DNA methylation at 3856 CpG sites across 126 immune‐related genes among ARFF firefighters. A few statistically significant associations between PFAS serum concentrations and epigenetic age were observed. Statistically significant decelerations in epigenetic age were associated with n‐PFOA according to four epigenetic age indicators: the Horvath, Hannum, PhenoAge, and skin‐blood clocks; and with PFUnDA according to the Hannum clock. In the primary analysis, only the association between n‐PFOA and the Skin‐Blood clock remained significant after Bonferroni correction. After exclusion of influential observations, the n‐PFOA/Skin‐Blood and PFUnDA/Hannum associations retained nominal significance but would not meet the same Bonferroni criterion. We report statistically significant associations (*q* < 0.1) between PFDA, n‐PFOA, Sm‐PFOS and DNA methylation of specific loci on 34 immune genes. Though we recommend results be interpreted cautiously due to relatively low serum concentrations of PFDA and PFUnDA, these data add to evidence of associations between certain long‐alkyl chain PFAS and the DNA methylome. These results contribute to understanding of potential mechanisms underlying PFAS immunotoxicity.

## Author Contributions

J.L.B., A.J.C.‐M., J.M.G., and M.M.C. designed the study. J.L.B. and A.J.C.‐M. applied for Research Ethics Board approval. P.L.‐F., S.C.B., R.K., R.M., and J.H. recruited the patients and collected the data. J.M.G., A.M.C., and J.C.B. generated the laboratory data. L.M.W. and J.M.G. analyzed the data. L.M.W. prepared the manuscript draft with important intellectual input from J.M.G. and M.F. All authors approved the final manuscript.

## Funding

This work was supported by Federal Emergency Management Agency, EMW‐2018‐FP‐00086; National Institute of Environmental Health Sciences, T32 ES007062; and National Institute for Occupational Safety and Health, T42 OH008455.

## Disclosure

The content is solely the responsibility of the authors and does not necessarily represent the official views of the NIH, the CDC, or NIOSH. Use of trade names is for identification only and does not imply endorsement by the CDC, the Public Health Service, or the United States Department of Health and Human Services.

## Conflicts of Interest

Dr. Jaclyn Goodrich has served as an expert witness for plaintiffs involved in litigation related to PFAS‐contaminated drinking water. The other authors declare no conflicts of interest.

## Supporting information


**Figure S1:** PRISMA diagram of literature search for gene inclusion.
**Figure S2:** Flow chart of sample selection process and inclusion criteria in statistical models.
**Figure S3:** Pearson correlations between PFAS serum concentrations and covariates.
**Table S1:** Immune genes included in the analysis.
**Table S2:** Sensitivity analysis. Associations between ln(PFAS) and Epigenetic Age Indicators (*n* = 177) after removal of influential data points. Adjusted for age, sex, race and ethnicity, smoking status, batch, and estimated cell type proportions for neutrophils, monocytes, NK cells, CD8 + CD28‐CD45RA‐ cells, CD4+ naive T‐cells, CD8+ naive T‐cells, CD4+ T‐cells, and plasmablasts. Associations with a *p*‐value < 0.05 are bolded and in red text.
**Table S3:** Loci significantly associated with multiple PFAS.

## Data Availability

The data that support the findings of this study are available on request from the corresponding author. The data are not publicly available due to privacy or ethical restrictions.
