# Supplementary material for: Associations of Serum Per‐ and Polyfluoroalkyl Substances (PFAS) With Epigenetic Age and DNA Methylation in US Airport Firefighters
Source: Environ Mol Mutagen. 2026 Oct 9;67(8-9):e70085. doi: 10.1002/em.70085 (PMC13653129; doi:10.1002/em.70085)
Supplement: Supplementary file 1 [file EM-67-0-s001.docx]

**Supplemental Material**

**Figure S1:** PRISMA diagram of literature search for gene inclusion

**Figure S2:** Flow chart of sample selection process and inclusion criteria in statistical models

**Figure S3.** Pearson correlations between PFAS serum concentrations and covariates

**Table S1:** Immune genes included in the analysis

| **Gene Names** | **Source Study and PFAS assessed** |
| --- | --- |
| *CCR5, CCR6, CD63, CSF2, CXCR4, GZMB, IFNG, IL12RB1, IL23R, IRF4, NFKB2, TNFSF15* | **(Maddalon et al., 2023) [1]**  *In vitro* PFAS treatment of primary human immune cells and gene expression analysis  PFBA, PFHxA, PFHxS, PFOA, PFOS, PFNA |
| *AADACL2, ADORA2A, ADAMTS20, AGR2, ANKRD40, ASF1A, CDC42BPB, CLUH, CETN2, CXorf23, CYTL1, DVL1, EGFEM1P, EMR4P, EPHB4, FAT1, GPR83, GPX8, HEPACAM2, HOXD8, IGSF11, IL27, INTS7, LOC100129361, LOC100131138, LOC100131298^, LOC100505711^, MAGEA8, MCF2L2, NUFIP1, ODF2, PCDHA11, PPARD, PRB2, PRSS36, RADIL, RAB3IP, RGS11, SCARB1, SCN1A, SHC4, STX18, SUPT16H, SYTL2, SUSD4, TRDN, TRIM42, VSTM2B, WISP1, ZKSCAN5*, ZNF615* | **(Pennings et al., 2016) [2]**  PFAS exposure assessment and cord blood gene expression analysis in the Norwegian BraMat cohort  PFOS, PFOA, PFNA, PFHxS |
| *CD40, CD8A, CR1, GYPC, HLA-DRB5, HLA-DQB1*, KIR2DL1, PF4, PPP1R11, RASL11B, RNF39, TAP2, TGFB3, THBS4* | **(Starling et al., 2020) [3]**  PFAS exposure assessment and cord blood DNA methylation analysis in Healthy Start Study  PFOA, PFOS, PFNA, PFDA, PFHxS |
| *CD19, EBF1, FCRLA, IGHM^, LAX1, POU2F2, RASGRP3, SLA, SPIB, TCF3, TCF4* | **(Rudzanová, et al., 2023)** [4]  PFAS exposure assessment and genome-wide gene expression analysis in PBMCs from the Central European Longitudinal Studies of Parents and Children: Young Adults  PFPA, PFOA, PFNA, PFDA, PFUnDA, PFHxS, PFOS |
| *ACSL4, ALAS1, PDK4, SLC25A6^, URM1, ZKSCAN5** | **(Ge et al., 2024)** [5]  Computational toxicology analysis of PFAS and gene expression data from placental samples  PFOA, PFOS, PFNA, PFHxS |
| *HCG9, HLA-DQB1*, LY6G5C, MPZL2, RNF39, TFCP2, ZNF416* | **(van den Dungen et al., 2017)** [6]  Persistent organic pollutant exposure assessment and blood DNA methylation analysis in Dutch men  PFOS |
| *ADAR, ANKHD1, C1S, C2, CFB, CCL2, CCL5, DDX60, GBP4, IFI35, IFIH1, IFIT1, IFIT3, IFIT5, IFI6, MX1, MX2, OAS1-3, OAS3, PTK2B, STAT1, SLC11A1, TLR3* | **(Chen et al., 2023) [7]**  *In vitro* PFOA treatment of human cytotrophoblasts and gene expression analysis  PFOA |
| *RAG1*, RAG2** | **(Janssen et al, 2022)** [8]  *In vitro* PFAS treatment of human Namalwa cells and gene expression analysis  PFOA, PFNA, PFHxS, PFOS |
| *IL2, RAG1*, RAG2** | **(Janssen et al., 2024) [9]**  *In vitro* PFAS treatment of human Namalwa cells and gene expression analysis  PFBA, PFHxA, PFOA, PFNA, PFDA, PFUnDA, PFBS, PFHxS, PFOS, 3:1 FTOH, 3:1 FTUOH, 4:2 FTOH, 6:2 FTOH, GenX, HFPO-TA |
| *APP, CCL8, DUSP19, GPD2, HS3ST3B1, IL32, KIF5B, LIME1, LRBA, SLFN12, TAX1BP1, TENM3, TNFAIP8L3* | **(Goodrich et al., 2021) [10]**  PFAS exposure assessment and blood DNA methylation analysis in the U.S. FFCCS  MeFOSAA, PFHxS, PFNA, PFDA, PFUnDA, n-PFOA, Sb-PFOA, n-PFOS, Sm-PFOS |
| *duplicates  ^dropped, no EWAS data | |

**Table S2:** Sensitivity analysis. Associations between ln(PFAS) & Epigenetic Age Indicators (n=177) after removal of influential data points. Adjusted for age, sex, race & ethnicity, smoking status, batch, and estimated cell type proportions for neutrophils, monocytes, NK cells, CD8+CD28-CD45RA- cells, CD4+ naive T-cells, CD8+ naive T-cells, CD4+ T-cells, and plasmablasts. Associations with a p-value<0.05 are bolded and in red text.

| *PFAS* | *Model Results* | Epigenetic Age Indicator | | | | | | |
| --- | --- | --- | --- | --- | --- | --- | --- | --- |
|  |  | *Horvath* | *Hannum* | *PhenoAge* | *SkinBlood* | *GrimAge* | *Dunedin PACE* | *Telomere length* |
| PFHxS | Estimate (SE) | 0.04  (0.44) | -0.17  (0.33) | -0.34  (0.50) | -0.41  (0.34) | 0.20  (0.25) | 0.002  (0.01) | 0.009  (0.01) |
|  | p-value | 0.93 | 0.62 | 0.50 | 0.24 | 0.43 | 0.85 | 0.55 |
| n-PFOS | Estimate (SE) | 0.07  (0.54) | -0.18  (0.40) | -0.48  (0.61) | 0.50  (0.42) | 0.15  (0.30) | 0.009  (0.01) | 0.004  (0.02) |
|  | p-value | 0.90 | 0.66 | 0.44 | 0.23 | 0.62 | 0.51 | 0.82 |
| Sm-PFOS | Estimate (SE) | -0.61  (0.50) | -0.50  (0.38) | -0.28  (0.57) | -0.22  (0.39) | 0.15  (0.28) | 0.01  (0.01) | 0.02  (0.02) |
|  | p-value | 0.23 | 0.18 | 0.62 | 0.58 | 0.60 | 0.41 | 0.18 |
| n-PFOA | Estimate (SE) | -1.00  (0.60) | -0.47  (0.45) | -0.63  (0.69) | **-1.00**  **(0.47)** | 0.11  (0.34) | -0.01  (0.02) | -0.10  (0.02) |
|  | p-value | 0.10 | 0.30 | 0.36 | **0.03** | 0.76 | 0.41 | 0.56 |
| PFNA | Estimate (SE) | -0.24  (0.54) | -0.68  (0.40) | -0.17  (0.62) | 0.11  (0.43) | 0.06  (0.31) | -0.007  (0.01) | -0.004  (0.02) |
|  | p-value | 0.66 | 0.10 | 0.78 | 0.80 | 0.85 | 0.61 | 0.83 |
| PFDA | Estimate (SE) | -0.07  (0.54) | -0.13  (0.40) | -0.34  (0.61) | 0.27  (0.42) | -0.03  (0.30) | -0.01  (0.01) | -0.01  (0.02) |
|  | p-value | 0.90 | 0.74 | 0.58 | 0.52 | 0.93 | 0.44 | 0.42 |
| PFUndA | Estimate (SE) | -0.15  (0.51) | **-0.79**  **(0.38)** | -0.70  (0.58) | -0.01  (0.40) | 0.14  (0.29) | -0.02  (0.01) | -0.02  (0.02) |
|  | p-value | 0.78 | **0.04** | 0.23 | 0.97 | 0.64 | 0.09 | 0.30 |
| MeFOSAA | Estimate (SE) | 0.54  (0.60) | 0.40  (0.45) | 1.11  (0.67) | 0.24  (0.47) | -0.35  (0.33) | -0.10  (0.10) | 0.02  (0.02) |
|  | p-value | 0.36 | 0.37 | 0.10 | 0.61 | 0.29 | 0.32 | 0.40 |

**Table S3:** Loci significantly associated with multiple PFAS

| **Probe ID** | **Gene Name** | **Gene Function** | **PFAS associated with this locus** | **Direction of association** |
| --- | --- | --- | --- | --- |
| cg03237507 | *DDX60* | Encodes an RNA helicase involved in antiviral innate immune responses and RIG-I-like receptor-mediated signaling | n-PFOA, PFDA | **↓** |
| cg17847344 | *EBF1* | Transcription factor with a role in B-cell differentiation and B-cell immune function | n-PFOA, PFDA | **↑** |
| cg25304359 | *MCF2L2* | Guanine nucleotide exchange factor involved in regulation of small GTPase signaling | n-PFOA, Sm-PFOS | **↑** |
| cg24192846 | *PPP1R11* | Inhibitor of protein phosphatase 1 (PP1), a phosphatase involved in regulation of cellular signaling processes | n-PFOA, PFDA | **↓** |
| cg23572228 | *RADIL* | Rap-binding signaling protein involved in cell adhesion and spreading; also regulates neutrophil adhesion and motility through integrin activation | n-PFOA, PFDA | **↑** |
| cg06971903 | *RASGRP3* | Guanine nucleotide exchange factor that activates RAS and RAP1 signaling; has also been implicated in immune signaling and systemic lupus erythematosus | n-PFOA, PFDA | **↓** |
| cg04141261 | *SCARB1* | Cell-surface receptor for HDL that mediates selective cholesterol and lipid transport between HDL and cells. This protein is also a receptor for hepatitis C virus glycoprotein E2 and facilitates cell entry by SARS-CoV2 | n-PFOA, PFDA | **↓** |
| cg07827972 |  |  | n-PFOA, PFDA | **↑** |
| cg13802355 | *SLC11A1* | Divalent metal transporter involved in iron and manganese transport, iron metabolism, and host resistance to infectious pathogens. | n-PFOA, PFDA | **↑** |
| cg06177974 | *SYTL2* | RAB27A-binding protein involved in RAB27A-dependent vesicle trafficking and intracellular cargo distribution | n-PFOA, PFDA | **↑** |

*Functional information obtained from NCBI Gene*

**References**

1.	Maddalon, A., et al., *Mixtures of per- and poly-fluoroalkyl substances (PFAS) reduce the in vitro activation of human T cells and basophils.* Chemosphere, 2023. **336**: p. 139204.

2.	Pennings, J.L., et al., *Cord blood gene expression supports that prenatal exposure to perfluoroalkyl substances causes depressed immune functionality in early childhood.* J Immunotoxicol, 2016. **13**(2): p. 173-80.

3.	Starling, A.P., et al., *Prenatal Exposure to Per- and Polyfluoroalkyl Substances, Umbilical Cord Blood DNA Methylation, and Cardio-Metabolic Indicators in Newborns: The Healthy Start Study.* Environ Health Perspect, 2020. **128**(12): p. 127014.

4.	Rudzanová, B., et al., *Altered Transcriptome Response in PBMCs of Czech Adults Linked to Multiple PFAS Exposure: B Cell Development as a Target of PFAS Immunotoxicity.* Environ Sci Technol, 2024. **58**(1): p. 90-98.

5.	Ge, Q., J. Zhao, and F. Qu, *Investigating the progression of preeclampsia through a comprehensive analysis of genes associated with per- and polyfluoroalkyl substances.* Toxicol Mech Methods, 2024: p. 1-10.

6.	van den Dungen, M.W., et al., *Association between DNA methylation profiles in leukocytes and serum levels of persistent organic pollutants in Dutch men.* Environ Epigenet, 2017. **3**(1): p. dvx001.

7.	Chen, H., et al., *Perfluorooctanoic Acid Induces Transcriptomic Alterations in 2nd Trimester Human Cytotrophoblasts.* Toxicol Sci, 2023.

8.	Janssen, A.W.F., et al., *Perfluoroalkyl substances (PFASs) decrease the expression of recombination-activating genes (RAG1 and RAG2) in human B lymphoma Namalwa cells.* Arch Toxicol, 2022. **97**(2): p. 457-68.

9.	Janssen, A.W.F., et al., *Determination of in vitro immunotoxic potencies of a series of perfluoralkylsubstances (PFASs) in human Namalwa B lymphocyte and human Jurkat T lymphocyte cells.* Front Toxicol, 2024. **6**: p. 1347965.

10.	Goodrich, J.M., et al., *Per- and polyfluoroalkyl substances, epigenetic age and DNA methylation: a cross-sectional study of firefighters.* Epigenomics, 2021.
